# Predictors of HIV testing among youth aged 15–24 years in The Gambia

**DOI:** 10.1371/journal.pone.0263720

**Published:** 2022-02-18

**Authors:** Ismaila Sonko, Min-Huey Chung, Wen-Hsuan Hou, Wei-Ti Chen, Pi-Chen Chang

**Affiliations:** 1 School of Nursing, College of Nursing, Taipei Medical University, Taipei, Taiwan; 2 Ministry of Health and Social Welfare, Banjul, The Gambia; 3 Department of Nursing, Taipei Medical University-Shuang Ho Hospital, New Taipei City, Taiwan; 4 Master Program in Long-Term Care, College of Nursing, Taipei Medical University, Taipei, Taiwan; 5 Graduate Institute of Clinical Medicine, College of Medicine, Taipei Medical University, Taipei, Taiwan; 6 Department of Education, Center of Evidence-Based Medicine, Taipei Medical University Hospital, Taipei, Taiwan; 7 School of Nursing, University of California, Los Angeles, California, United States of America; University of Salamanca, SPAIN

## Abstract

**Background:**

Worldwide, an estimated 38.0 million people lived with the human immunodeficiency virus in 2019, and 3.4 million young people aged 15~24 years were living with HIV. Sub-Saharan Africa carries a significant HIV burden with West and Central Africa most affected with HIV. Among the young people living with HIV in West and Central Africa, an estimated 810,000 were aged 15~24 years. This study aimed to assess predictors that influence the uptake of HIV testing among youth aged 15~24 years in The Gambia.

**Methods:**

The 2013 Gambia Demographic and Health Survey data for youth aged 15~24 years was used. The Andersen behavioral model of health service use guided this study. A cross-sectional study design was used on 6194 subjects, among which 4730 were female. The analysis employed Chi-squared tests and hierarchical logistic regression.

**Results:**

Less than one-quarter of the youth 1404 (22.6%) had ever been tested for HIV. Young people aged 20~24 years (adjusted odds ratio (aOR): 1.98), who were females (aOR: 1.13), married youth (aOR: 3.89), with a primary (aOR: 1.23), secondary or higher education (aOR: 1.46), and who were from the Jola/Karoninka ethnic group (aOR: 1.81), had higher odds of having been tested for HIV. Those with adequate HIV knowledge and those who were sexually active and had aged at first sex ≥15 years (aOR: 3.99) and those <15 years (aOR: 3.96) were more likely to have been tested for HIV compared to those who never had sex.

**Conclusion:**

This study underscores the low level of model testing on HIV testing among youth (15~24 years) in The Gambia. Using Anderson’s Model of Health Service Utilization, the predisposing factors (socio-demographic and HIV knowledge) and the need-for-care factors (sexual risk behaviors) predict healthcare utilization services (HIV testing) in our study; however, only socio-demographic model explained most of the variance in HIV testing. The low effect of model testing could be related to the limited number of major variables selected for HIV knowledge and sexual risk behavior models. Thus, consideration for more variables is required for future studies.

## Introduction

Globally, an estimated 38.0 million people lived with the human immunodeficiency virus (HIV) in 2019 [1], and 3.4 million young people aged 15~24 years were living with HIV [2]. Sub-Saharan Africa (SSA) carries a significant HIV burden, and West and Central Africa were the most affected regions with HIV. Among the young people living with HIV (PLWHIV) in West and Central Africa, an estimated 810,000 were aged 15~24 years [3]. The Gambia has also been affected by the HIV epidemic, with an estimated 1761 (0.4%) adolescents aged 15~19 years and 1686 (0.1%) youth aged 20~24 years with HIV [4]. Despite the proportion of PLWHIV in SSA, adolescents had not benefited as substantially from HIV responses compared to other regions; hence a sense of urgency for this vulnerable population seems to be lacking [3].

HIV testing is a critical entry point for prevention, care, and treatment, as it facilitates early diagnosis and enhances prompt treatment to reduce new infections among vulnerable youth populations [5, 6]. HIV testing is also essential in preventing mother-to-child transmission of HIV and preventing pregnancy among adolescents [3]. The Joint United Nations Program on AIDS/HIV (UNAIDS) launched an ambitious 90-90-90 target towards ending the HIV epidemic by 2030, with the aim that by 2020, 90% of PLWHIV will know their status, 90% of those diagnosed with HIV infection will receive sustained antiretroviral therapy (ART), and 90% of those on treatment will have suppressed viral loads [7]. The 2020 Global HIV Statistics uncovered that estimated 84% of PLWHIV knew their HIV status [2]. HIV testing uptake still remain low in SSA. Among PLWHIV, the majority (65%) of young people aged 15~24 years did not know their HIV status [8]. If the current trend continues, hundreds of thousands more will become HIV positive in the coming years, and without knowing their status, adolescents will miss out on life-saving treatment [9].

The major significant predisposing factors in utilizing healthcare treatment services in SSA include age, marital status, gender, educational level, place of residence, and ethnicity [10–12]. Age has a significant association with testing among youth in SSA, and HIV testing was lower in adolescents (15~19 years) than those aged 20~24 [11–13]. Marital status and gender showed an association with HIV testing [14–18]. The odds of testing were higher among married youth compared to single youth [11, 16]. Evidence showed that in Zambia, Nigeria, and South Africa, females were most likely than males to get tested for HIV [15, 19, 20]. Youth who had received a formal education were more likely to be tested than those with no education [11, 12, 18, 19], higher in urban dwellers [15, 21, 22]. The association of ethnicity and HIV testing was less studied in SSA; however, race/ ethnicity has significantly predicted HIV testing in the United States (US). The black youth population in the US had lower odds of getting tested than white folks [23], contrary to similar US findings [24].

HIV knowledge was also a significant predisposing factor for testing among youth [14, 19]. Studies across countries in SSA found a positive relationship between HIV knowledge and HIV testing. Youth in Kenya who had HIV knowledge were 1.19-times more likely to be tested for HIV [25], and a similar finding was drawn from a study done in Nigeria where youth with higher HIV knowledge were 1.62-times more likely to be tested [19].

Sexual risk behaviors (SRB) were positively associated with healthcare services utilization (HIV testing) among youth [12, 13, 15, 17, 26]. The association between age at sexual debut and HIV testing was reported in many studies in SSA. Evidence showed that youth who were older at sexual debut (15~18 years, or 19 years and above) had higher odds of HIV testing (aOR = 1.22; 1.07–1.39 and aOR = 1.47; 1.24–1.74) than those who had age at sexual debut <15 years [17], and in Malawi, men who had their first sexual debut at 15~24 years had higher odds of being tested (AOR = 2.54; 95% CI = 2.11–3.07) than men who never had sex [13]. A similar conclusion was drawn from a study done in Zambia where youth at an older age (M = 15.22, SD = 1.746) compared to younger age were more likely to be tested for HIV than those that had not tested (M = 14.91, SD = 1.820) [15]. There are mixed results from other studies regarding condom use and HIV testing uptake. Testing was high among male youth who had not used a condom in Tanzania and KwaZulu Natal, South Africa [16]. Contrarily, in Nigeria, youth who had used a condom were more likely to be tested for HIV [19]. Studies suggested that young women with multiple sexual partners (MSPs) had higher odds of being tested for HIV [22].

The selection of variables to be added in our model was guided by the Anderson’s Behavioral Model of health service use [27] to examine the predictors of HIV testing service among youth aged 15~24 years in The Gambia. The Anderson Behavioral Model of health service utilization is one example of numerous theories that provide a valuable framework to understand the underlying population characteristics (predisposing and need-for-care factors) that influence health service use [28]. The model suggests that health outcome such as Personal Health Practice is influenced directly by population characteristics (predisposing characteristics and the need components) as shown in S1 Fig. The critical elements in the model consist of the predisposing factors, the need factors that influence the utilization of healthcare services by individuals [29]. The predisposing factors are two folds. First, socio-demographic factors include age, marital status, gender, educational level, wealth index, residence, ethnicity, and the second predisposing factor includes HIV knowledge. At the same time, we examined the sexual risk behaviors as the ‘need-for-care’ components. HIV testing services were assessed using the personal health practice as health outcome for youth. This model has been validated and applied in previous HIV testing studies in Thailand, Haiti, Ghana, and the US [29–34]. The model is considered appropriate for our study since it is a multilevel theory and has been applied in many settings and disciplines [32].

Most previous studies in SSA, including The Gambia, have focused on HIV testing services among adult and pregnant women but have not focused on adolescents and youth. The present study expands upon the previous studies by focusing on youth utilization of HIV testing services to identify any existing gap as demonstrated in previous studies [5, 35–37]. Based on our best knowledge, no study has been conducted on HIV testing that used the Anderson’s Behavioral Model on adolescents and youth in The Gambia. This study aimed to assess predictors that influence the uptake of HIV testing among youth aged 15~24 years in The Gambia.

## Materials and methods

### Design

A cross-sectional study design was used in this study.

### Sample

The data used for the study was extracted from the 2013 Gambia Demographic and Health Survey (GDHS) [4]. A total of 6194 youth aged 15~24 years took part in this analysis, among which 4501 were females. In 2013, The Gambia had a total population of 1.9 million. Women constituted 51% of the total population, and about 22% were aged 15~24 years. An estimated 50% of the country’s residents live in rural areas, and the literacy rate, which decreases with an increase in age in both sexes, was higher among men than women (70% vs. 45%). The Gambia is a multilingual country with more than six ethnic groups, and the two most widely known religions are Islam and Christianity. The vast majority of both women and men (96%) are Muslims, 4% believe in Christianity, and a small proportion (<1%) claimed to have no religion [4].

### Data collection

The 2013 GDHS is a nationally representative household survey conducted in The Gambia through the Gambia Bureau of Statistics (GBOS). The GDHS is a nationwide DHS as part of the worldwide DHS that includes more than ninety countries, funded by the United States Agency for International Development [4]. A nationally representative survey uses a multistage and stratified design to collect population health, HIV/AIDS, malaria, and nutrition information. Due to discrepancies in samples across settings, sample weights were applied to compensate for the unequal probability of selection between the geographically defined strata and non-responses. A comprehensive explanation of the weighting procedure can be accessed in a DHS methodology report [4]. The current study considered both sexually active and non-sexually active youth aged 15~24 years about the discrepancies in samples across settings after applying sample weights to recompense for the unequal probability of selection between the geographically defined strata and non-responses [4].

### Validity and reliability/rigor

Three questionnaires were included in the 2013 GDHS: the Household Questionnaire, the Woman’s Questionnaire, and the Man’s Questionnaire. The 2013 GDHS questionnaires have their content validity that they were based on the models developed by the Demographic and Health Survey (DHS) program with modifications to accommodate The Gambia’s specific needs, according to discussions between ICF International and staffs from various governmental institutions, non-governmental organizations, donors, and development partners. Hence, the survey contains questions that cover all aspects of the Gambia population health measured.

Ever been tested for HIV was used as the outcome for healthcare utilization services in this study, coded (0) for "No" if respondents had never been tested for HIV and (1) for "Yes" if respondents had been tested for HIV. The final sample size used in this study arrived at 6194 from 6220 after we employed multiple imputations to handle missing data. The predisposing factors for socio-demographic characteristics were defined as follows: age was categorized into two levels, 15~19 and 20~24 years. Gender defined as male coded (0) and female coded (1); educational level: no education (0), primary (1), and secondary/higher education (2); wealth; poorest (0), poorer (1), poor (2), richer (3), and richest (4); place of residence: urban (0) and rural (1); and ethnicity defined as Mandinka/Jahanka (0), Wollof (1), Jola/Karoninka (2), Fula/Tukulur/Lorobo (3), Serere (4), Sarahuleh (5), non-Gambian (6), others (7). The predisposing factors for HIV knowledge variables included (1) "a healthy person can get HIV", (2) "a person can get HIV through witchcraft", (3) "a person can get HIV by sharing food with an infected person", and (4) can condom use every time during sex reduce the risk of HIV, using the prefix ’correct’ (1) or ’incorrect’ (0). The need-for-care factors for SRB variables include (1) Age at first sex, using the prefix ‘never had sex (0), age <15 (1), and ≥15 years (2). (2) Condom use during sex in the last 12 months, (3) had any sexually transmitted infections (STIs) in the previous 12 months, and (4) having MSPs using the prefix 1 for ’yes’ and 0 for ’no’ based on the respondent’s answers.

### Ethical considerations

Permission to use 2013 GDHS data was sought from the Inner-City Fund (ICF) International (530 Gaither Road, Suite 500 Rockville, MD 20850, USA) and the institutional review board (IRB) committee of Taipei Medical University (Taipei, Taiwan).

### Statistical analysis

Descriptive statistics, Chi-squared comparisons, and hierarchical logistic regressions were employed to determine predictors for HIV testing. Extracted data for youth were weighted as representative of 15~24year-old respondents in the 2013 GDHS. All analyses performed used the Statistical Package for the Social Sciences (SPSS) vers. 21.

We employed multiple imputations (MI) using SPSS to deal with missing responses on variables from the selected potential predictor variables. Among the predictors that have missing data include ethnicity (0.5%), a healthy person can have HIV (0.4%), a person can have HIV through witchcraft (0.1%), a person can have HIV by sharing food with an infected person (0.1%), can condom use every time during sex reduce the risk of HIV (0.2), age at first sex (0.1%), condom use during sex in the past 12 months (8.6%), having STI in the past 12 months (0.2%), and had Multiple Sexual Partners (0.1%) (Table 1).

**Table 1:** Demographic characteristics, HIV knowledge, and behaviour (n = 6220).

Variable	Frequency		Missing n(%)
	n	(%)	
***Socio-demographics***			
Age (Years)			
15–19	3330	53.5	
20–24	2890	46.5	
Marital Status			
Single	4162	66.9	
Married	2058	33.1	
Gender			
Male	1490	24.0	
Female	4730	76.0	
Educational level			
No education	1789	28.8	
Primary	1074	17.3	
Secondary/Higher	3357	54.0	
Wealth index			
Poorest	1203	19.3	
Poorer	1365	21.9	
Poor	1202	19.3	
Richer	1097	17.6	
Richest	1353	21.8	
Residence			
Urban	2857	45.9	
Rural	3363	54.1	
Ethnicity			31(0.5)
Mandinka/Jahanka	2090	33.6	
Wollof	823	13.2	
Jola/Karoninka	484	7.8	
Fula/Tukulur/Lorobo	1551	24.9	
Others	1241	20.0	
***HIV Knowledge***			
Can a healthy person have HIV			22(0.4)
Incorrect	2689	43.2	
Correct	3509	56.4	
Can a person get HIV through witchcraft			8(0.1)
Incorrect	1658	26.7	
Correct	4554	73.2	
Can a person get HIV by sharing food with an infected person			7(0.1)
Incorrect	1950	31.4	
Correct	4263	68.5	
Can condom use every time during sex reduce the risk of HIV			13(0.2)
Incorrect	1912	30.7	
Correct	4295	69.1	
***Sexual Risk Behavior***			
Age at first sex			5(0.1)
Never had sex	3468	55.8	
< 15 years	117	1.9	
≥ 15 years	2630	42.3	
Did you use condom in the last 12 months			533(8.6)
No	5437	87.4	
Yes	250	4.0	
Had any STI in the last 12 months			15(0.2)
No	6161	99.1	
Yes	44	0.7	
Had Multiple Sexual Partners			7(0.1)
No	6164	99.1	
Yes	49	0.8	
Have you ever been tested for HIV			26(0.4)
No	4790	77.0	
Yes	1404	22.6	

Hierarchical regression was used as a framework for model comparison in our study. We considered a Pseudo-R-squared, which are standard measures for investigating the goodness of fit of logit models used to evaluate single models and compare different models to identify the model with the best fit in the logistic regression. McFadden pseudo-R-squared was considered in this study. McFadden pseudo-R-squared for the logistic regression model of 0.35 depicts a fair model predictive power (The McFadden pseudo-R-squared measure ranges from 0 to below 1, with values closer to 0 indicating lack of predictive power) [38].

The Akaike Information Criterion (AIC) goodness-of-fit test to identify the model fitted and AIC can help achieve the trade-off required between simplicity and adequacy of model fitting [39, 40]. HIV testing among youth in The Gambia from a class of different models such as; socio-demographic (model I), HIV knowledge (model II), and sexual risk behavior (model III) was used. For AIC, the smaller indicates a more parsimonious model relative to a model fit with larger AIC. The adjusted odds ratios and 95% confidence intervals were given, with the alpha level set to 0.05.

## Results

### Characteristics of study participants

The respondents’ predisposing factors (socio-demographic characteristics and HIV knowledge), and the need-for-care factors (sexual risk behaviors) were presented in Table 1. A total of 6194 participants took part in this study. The majority consisted predominantly of youth aged 15~19 years 3330 (53.5%), single 4162 (66.9%), and females 4730 (76.0%). About half, 3357 (54.0%) of the respondents had at least a secondary/higher education, the majority fell within both the poorer 1365 (21.9%) and richest categories 1353 (21.8%), and slightly more than half 3363 (54.1%) lived in rural areas. Higher percentages were from the Mandinka/Jahanka 2090 (33.6%) and Fula/Tukulur/Lorobo 1551 (24.9%) ethnic groups. Regarding HIV knowledge, more than half 3509 (56.4%) had adequate knowledge that a healthy person can have HIV. A similar trend on increased HIV knowledge was demonstrated for the following HIV knowledge variables: a person gets HIV through witchcraft, a person gets HIV by sharing food with an infected person, and condom use every time during sex reduce the risk of HIV (4554, (73.2%), 4263 (68.5%), 4295 (69.1%)), respectively. The study further revealed that SRB variables include age at first sex, condom used, had any STIs in the past 12 months, and having multiple sexual partners. Few 117 (1.9%) demonstrated age at first sex at aged <15 years, more than half 3468 (55.8%) never had sex, and 2630 (42.3%) experienced their first sex at age ≥15 years. Only a few had ever used condom 250 (4.0%) compared to those who had not used a condom 5437 (87.4%), about 6161 (99.1%) had no history of STIs, 49 (0.8%) had MSPs, and only a limited proportion 1404 (26.6%) had ever been tested for HIV.

### Bivariate analysis of predictors of HIV testing among youth

Table 2 presents the relationship between socio-demographic characteristics, HIV knowledge, sexual risk behaviors, and uptake of HIV testing among youth in The Gambia. An estimated 3468 (55.8%) of youth were not sexually active, of whom 237 (16.9%) were ever tested for HIV. Overall, only 1404 (22.6%) had ever been tested for HIV across socio-demographic, HIV knowledge, and SRB strata. Having been tested for HIV was more common among youth aged 20~24 years than adolescents 15~19 years (1024 (72.9%) vs. 380 (27.1%)), *p*<0.001, married respondents than single ones (993 (70.7%) vs. 411 (29.3%)), *p*<0.001, among females than males (1090 (77.6%) vs. 314 (22.4%)), *p*<0.001, and among those with a secondary/higher education than those with a primary or no formal education (670 (47.7%) vs. 230 (16.4% or 504 (35.9%))), *p*<0.001. HIV testing was statistically significant in all four HIV knowledge variables such as a healthy person can have HIV (*p* = 0.001), a person gets HIV through witchcraft (*p* = 0.005), a person gets HIV by sharing food with an infected person (*p*<0.001), and condom use every time during sex reduce the risk of HIV (*p*<0.005). Regarding behaviors, HIV testing was high among those who delayed their first sexual debut. The majority of those tested for HIV were aged ≥15 years compared to those who initiated sex at age <15 years and those who never had sex (*p*<0.001). Among youth who reported a history of STI, 22 (1.6%) had tested for HIV compared to 98.4% of youth with no history of STI (*p*<0.001).

**Table 2:** Correlation of socio-demographic, HIV knowledge, behaviour and HIV testing (n = 6194).

Variable	Ever been tested for HIV		
	No (n = 4790)	Yes (n = 1404)	
	n (%)	n (%)	*P*-value
***Socio-demographics***			
Age (years)			0.000**
15–19	2943 (61.4)	380 (27.1)	
20–24	1847 (38.6)	1024 (72.9)	
Marital Status			0.000**
Single	3746 (78.2)	411 (29.3)	
Married	1044 (21.8)	993 (70.7)	
Gender			0.105
Male	1172 (24.5)	314 (22.4)	
Female	3618 (75.5)	1090 (77.6)	
Educational level			0.000**
No education	1272 (26.6)	504 (35.9)	
Primary	841(17.6)	230 (16.4)	
Secondary/Higher	2677 (55.9)	670 (47.7)	
Wealth index			0.246
Poorest	926 (19.3)	267 (19.0)	
Poorer	1032 (21.5)	327 (23.3)	
Poor	924 (19.3)	274 (19.5)	
Richer	836 (17.5)	258 (18.4)	
Richest	1072 (22.4)	278 (19.8)	
Place of residence			0.228
Urban	2223 (46.4)	626 (44.6)	
Rural	2567 (54.0)	778 (55.4)	
Ethnicity			0.067
Mandinka/Jahanka	1643 (34.3)	447.2(31.9)	
Wollof	637.8(13.3)	188.8(13.4)	
Jola/Karoninka	348.2(7.3)	132 (9.4)	
Fula/Tukulur/Lorobo	1208 (25.2)	344.2(24.5)	
Others	953 (19.9)	291 (20.7)	
***HIV Knowledge***			
Can a healthy person have HIV			0.001*
Incorrect	2132 (44.5)	556 (39.6)	
Correct	2658 (55.5)	848 (60.4)	
Can a person get HIV through witchcraft			0.005*
Incorrect	1316 (27.5)	332 (23.6)	
Correct	3474 (72.5)	1072 (76.4)	
Can a person get HIV by sharing food with an infected person			0.000**
Incorrect	1567 (32.7)	373 (26.6)	
Correct	3223 (67.3)	1031 (73.4)	
Can condom use every time during sex reduce risk of HIV			0.008*
Incorrect	1514 (31.6)	393 (28.0)	
Correct	3276 (68.4)	1011 (72.0)	
***Sexual Risk Behavior***			
Age at first sex			0.000**
Never had sex	3233 (67.5)	237 (16.9)	
< 15 years	86 (1.8)	29 (2.1)	
≥ 15 years	1471 (30.7)	1138 (81.2)	
Did you use condom use in the last 12 months			0.110
No	4497 (93.9)	1300 (92.6)	
Yes	293 (6.1)	104 (7.4)	
Had any STI in the last 12 months			0.000**
No	4768 (99.5)	1382 (98.4)	
Yes	22 (0.5)	22 (1.6)	
Had Multiple Sexual Partners			0.821
No	4752 (99.2)	1392 (99.1)	
Yes	38 (0.8)	12 (0.9)	

*p < 0.05

**p < 0.001.

Tested by Chi-Square.

### Multivariate analysis of predictors of HIV testing among youth

Results from adjusted logistic regression models are presented in Table 3. Positive associations were evident between HIV testing and the following variables: age, marital status, educational level, ethnicity, a healthy person can have HIV, a person gets HIV by sharing food with an infected person, and age at sexual debut.

**Table 3:** Multivariate hierarchical logistic regression of HIV testing (n = 6194).

Variable	Model I			Model II			Model III		
	aOR	95% CI	*P*-value	aOR	95% CI	*P*-value	aOR	95% CI	*P*-value
***Socio-demographics***									
Age (Years)									
15–19	Ref	-	-	-	-	-	-	-	-
20–24	2.50	2.32–2.69	0.000**	2.39	2.22–2.58	0.000**	1.98	1.70–2.30	0.000**
Marital status									
Single	Ref	-	-	-	-	-	-	-	-
Married	9.25	8.51–10.05	0.000**	9.41	8.04–11.01	0.000**	3.89	3.07–4.92	0.000**
Gender									
Male	Ref	-	-	-	-	-	-	-	-
Female	1.10	1.01–1.21	0.258	1.11	0.92–1.32	0.255	1.13	0.95–1.34	0.187
Educational level									
No education	Ref	-	-	-	-	-	-	-	-
Primary	1.29	1.04–1.59	0.019*	1.24	1.01–1.54	0.045*	1.23	1.00–1.52	0.059
Secondary/Higher	1.51	1.26–1.81	0.000**	1.39	1.15–1.67	0.001*	1.46	1.21–1.76	0.000**
Wealth index									
Poorest	Ref	-	-	-	-	-	-	-	-
Poorer	1.06	0.87–1.30	0.599	1.05	0.85–1.30	0.673	1.06	0.85–1.31	0.610
Poor	1.02	0.81–1.27	0.885	1.00	0.80–1.25	0.987	1.02	0.81–1.27	0.887
Richer	1.06	0.80–1.40	0.683	1.04	0.78–1.37	0.801	1.10	0.83–1.46	0.514
Richest	1.00	0.75–1.34	0.998	0.95	0.70–1.27	0.719	1.04	0.77–1.41	0.787
Place of residence									
Rural	Ref	-	-	-	-	-	-	-	-
Urban	0.78	0.62–0.97	0.027*	0.80	0.63–1.00	0.050	0.83	0.66–1.04	0.100
Ethnicity									
Mandinka/Jahanka	Ref	-	-	-	-	-	-	-	-
Wollof	1.08	0.86–1.35	0.523	1.11	0.88–1.39	0.389	1.11	0.88–1.40	0.390
Jola/Karoninka	1.97	1.51–2.56	0.000**	1.00	1.53–2.60	0.000**	1.81	1.38–2.37	0.000**
Fula/Tukulur/Lorobo	0.96	0.80–1.16	0.698	1.00	0.83–1.21	0.989	1.00	0.83–1.21	0.993
Others	1.08	0.89–1.32	0.436	1.10	0.90–1.34	0.339	1.04	0.85–1.27	0.697
***HIV Knowledge***									
Can a healthy person have HIV									
Incorrect				Ref	-	-	-	-	-
Correct				1.23	1.06–1.43	0.005*	1.22	1.05–1.42	0.008*
Can a person get HIV by witchcraft									
Incorrect				Ref	-	-	-	-	-
Correct				1.11	0.933–1.324	0.236	1.09	0.91–1.30	0.344
Can a person get HIV by sharing food with an infected person									
Incorrect				Ref	-	-	-	-	-
Correct				1.23	1.04–1.45	0.015*	1.26	1.06–1.49	0.007*
Can condom use every time during sex reduce risk of HIV									
Incorrect				Ref	-	-	-	-	-
Correct				1.15	0.98–1.35	0.090	1.12	0.95–1.31	0.184
***Sexual Risk Behavior***									
Age at first sex									
Never had sex							Ref	-	-
< 15 years							3.96	2.46–6.39	0.000**
≥ 15 years							3.99	3.11–5.13	0.000**
Did you use condom use in the last 12 months									
Yes							Ref	-	-
No							0.74	0.54–1.02	0.062
Had any STI in the last 12 months									
No							Ref	-	-
Yes							1.28	0.67–2.43	0.458
Had multiple sexual partners									
No							Ref	-	-
Yes							0.83	0.42–1.67	0.608
**McFadden Pseudo R** ^**2**^	0.21			0.22			0.24		

*p < 0.05

**p < 0.001; aORs ~ adjusted Odds Ratio; CI ~ Confidence Interval; Model I ~ socio-demographic factors; Model II ~ socio-demographic factors + HIV Knowledge related factors; Model III ~ socio-demographic factors + HIV knowledge related factors + sexual risk behavior related factors; R^2^ ~ R-squared.

The multivariate analysis of our result includes three regression models. Model I consists of only the sociodemographic characteristics that were statistically significantly associated with HIV testing. The significant sociodemographic variables include age = aOR 2.50, (95% CI = 2.32–2.69), marital status = aOR 9.25, (95% CI = 8.51–10.05), educational level (Primary = aOR 1.29, (95% CI = 1.04–1.59); Secondary/Higher = aOR 1.51, (95% CI = 1.26–1.81)), and ethnicity (Jola/Karoninka = aOR 1.97, (95% CI = 1.51–2.56)).

A similar regression analysis demonstrated in Model II for HIV knowledge variables such as: a healthy person can get HIV, a person can get HIV through witchcraft, a person can have HIV by sharing food with an infected person, condom use every time during sex reduce risk of HIV. Only a healthy person can get HIV (aOR = 1.23, (95% CI = 1.06–1.43)) and a person can have HIV by sharing food with an infected person (aOR = 1.23, (95% CI = 1.04–1.45)) were significantly associated with HIV testing, Table 3.

In model III, youth aged 20~24 years had higher odds of HIV testing than youth aged 15~19 years (adjusted OR (aOR) = 1.977; (1.696–2.304)). Married youth were 3.885-times more likely to be tested for HIV (95% CI = 3.065–4.924) compared to single youth, p<0.001, and youth who had secondary/higher education were 1.455 times (95% CI = 1.207–1.755) more likely to be tested for HIV than those who had primary or no education, p<0.001. Youth from the Jola/Karoninka tribes compared to the Mandinka/Jahanka tribes (aOR = 1.810, 95% CI = 1.381–2.373), p<0.001. Also, those with adequate knowledge of HIV had higher odds of being tested for HIV than youth without adequate HIV knowledge. The significant variables that indicated adequate HIV knowledge such as, a healthy person can have HIV, a person gets HIV by sharing food with an infected person had higher odds of been tested for HIV compared to those who had low HIV knowledge (aOR = 1.221, (1.053–1.417)) and (aOR = 1.259, (1.064–1.491)). Compared to youth who were not sexually active, those age at sexual debut <15 years, aOR = 3.960, (2.455–6.387), those who were older at sexual debut (≥15 years) had higher odds of HIV testing (aOR = 3.994; (3.108–5.134)).

In this study, the McFadden Pseudo R-squared statistic results from the baseline individual socio-demographic characteristic indicated an R^2^ score of 0.211 (21.0%) for model I, 0.22 (22.0%) for model II, and 0.24 (24.0%) model III, respectively. The McFadden Pseudo R-squared statistical test result has slightly increased from 21.0% to 22.0% after included HIV knowledge in the equation. The behavior variables (model III) had slight increase from 22.0% to 24.0% in the equation than model II. Therefore, our results indicated that both HIV knowledge and SRB variables significantly impacted HIV testing uptake among young people.

The Akaike Information Criterion (AIC) goodness-of-fit test to identify the model fitted for HIV testing from a class of different models such as; socio-demographic (model I), HIV knowledge (model II), and sexual risk behavior (model III) was used. For final discussion, the following AIC results were obtained for models I, II, and III, 0.252, 0.583, and 0.622, respectively. Among the three models tested in our study, model I (socio-demographic) demonstrated a better model fit than model II (HIV knowledge) and model III (sexual risk behavior).

## Discussion

Employing the healthcare service utilization model to explain HIV testing, our results indicated a low proportion of youth (15~24 years) ever tested for HIV, particularly among male adolescents. Only 1404 (22.6%) youth had ever tested for HIV compared to the overall proportion of Gambians (36.0%) ever tested [4]. This findings are still farfetched from the 90% target of UNAIDS 2020 ambitious goals to end the HIV epidemic by 2030 [4, 7, 41]. Our results corroborate other SSA studies, including Congo, Mozambique, and Nigeria (31.4%, 45.3%, and 24.7%), respectively [17]. Low HIV testing among youth could be related to the limited access to HIV testing services and the lack of trust in HIV testing services [42]. Other barriers to testing include fear of stigmatization, fear of a positive diagnosis, the perceived risk concerning sexual exposure, and poor attitudes by healthcare professionals [43]. A study suggests that most interventions to raise HIV testing uptake focused more on antenatal women [44], while the highly at-risk young populations had minimal attention [45]. Thus, it is essential to engage policymakers to develop a policy framework to improve access and uptake of HIV testing services among adolescents and youth [19].

The predisposing factors such as age, marital status, educational level, and ethnicity demonstrated a statistically significant association with HIV testing in this study. The current study found that older youth aged 20~24 years had higher odds of HIV testing than did adolescents (15~19 years). This result is considered consistent across other SSA studies [11, 12, 19, 46]. The first reason could be that older youth aged 20~24 are more likely to be sexually active, more likely to be married, and more likely to be economically empowered and knowledgeable on HIV issues than youth aged 15~19 [46, 47], and youth aged 15~19 have a lower self-perceived likelihood of HIV [48]. Second, youth aged 20~24 years tend to have more lifetime exposure to HIV testing services and are more likely to access healthcare facilities through several programs, including testing during reproductive health services [12]. School-based HIV/AIDS educational interventions and adolescent-friendly service initiation could promote positive SRBs and increase testing uptake [31, 49].

Marital status was significantly associated with HIV testing among youth in this study, with married youth (aOR 3.89) having higher odds of HIV testing than single youth. Our results showed consistent findings with other SSA studies [11, 15, 18, 46]. The reason for increased odds of HIV testing among married youth compared to single youth could be the HIV testing services offered during ANC visits as suggested in studies that adolescents and young women who had attended ANC or had given birth in the health facility have greater chances of getting tested as testing is a prerequisite for antenatal women at certain SSA countries [15, 19, 37, 46].

The odds of being tested for HIV increase with an increase in the educational level, corroborating other SSA studies [11, 13, 18, 19]. Attaining formal education improves HIV knowledge, increases youth health literacy levels, and facilitates relevant decision-making enhancing health facility visits and health services utilization [11, 13]. A previous study outlined gaps in educational levels among adolescents, and filling these gaps will require concerted efforts [50]. By using community radio widely popular among youth in The Gambia, prominent people in the community, including traditional and faith leaders and civil society organizations, could enlighten adolescents and youth with low literacy and low utilization of healthcare services [4, 12, 51].

Ethnic disparity had a significant positive association with HIV testing among youth in The Gambia, especially among the Jola/Karoninka ethnic group. Previous studies conducted in Ghana and South Africa found ethnicity as a significant predictor of HIV testing [32, 46]. The rate of HIV testing uptake among the Jola/Karoninka ethnic group in The Gambia remained positively associated with their strong socio-cultural beliefs. However, socio-cultural factors have negatively influenced HIV testing uptake among the Mole-Dagbani ethnic group in Ghana [32]. The socio-cultural differences between Ghana and The Gambia have demonstrated ethnic disparities on HIV testing uptake that may require future qualitative studies.

HIV knowledge is also a predisposing factor derived from the Anderson’s Behavioral Model used in our study. In this study, youth with adequate HIV knowledge had higher testing odds than previous studies [14, 19, 25, 52]. Respondents who had adequate HIV knowledge that a healthy person can get HIV and a person get HIV by sharing food with an infected person were 1.22 and 1.26-times more likely to have been tested for HIV than those without adequate HIV knowledge. Despite increased HIV knowledge among youth, the misconception surrounding a healthy person having HIV and sharing food with an infected person still exists [4].

Age at first sex is the only significant variable of the need-for-care factor associated with HIV testing in the current study, as confirmed with previous studies in SSA [13, 15, 17, 53]. In our study, youth who were sexually active had higher odds of HIV testing than non-sexually active youth. Our finding was supported by other studies in SSA, such as Malawi, Zambia, and a few countries that represent Central, South, East, and West Africa [13, 15, 17]. More attention should be shifted to non-sexually active youth who had lower odds of HIV testing to be educated more on HIV testing and utilize available healthcare services. Healthcare workers should make HIV test information more accessible for youth with high SRBs since most countries in Africa have low HIV testing coverage and miss a rare opportunity for positive prevention [53].

This study sought to examine the predictors of HIV testing services among youth based on Anderson’s behavioral model [27]. The overall model testing for the predisposing factors (socio-demographic and HIV knowledge) and the need-for-care factors (sexual risk behaviors) as predictors to healthcare service utilization (HIV testing) were found low in this study, especially for models II and III. The low explained variance may indicate that essential predictors lack from the prediction model.

Despite the poor model fit of models II and III, our result finds that the socio-demographic as predisposing factors explained most of the variance in HIV testing. Age, marital status, educational level, and ethnicity were the significant predictors of HIV testing consistent with previous studies [19, 30, 32, 54].

HIV knowledge is a predisposing factor associated with HIV testing. Our finding is consistent with results in Ethiopia which argued that lack of comprehensive HIV knowledge decreases the odds of adolescents and youth uptake of HIV testing services [54]. Previous studies suggest that comprehensive HIV knowledge could positively affect HIV testing [17, 55]. A recent UNICEF report demonstrates that HIV knowledge among adolescents in The Gambia is at the lowest compared to other countries, even much lower than other SSA countries, which should be a considerable concern for policymakers [9]. Knowledge of HIV/AIDS includes four items: one knowledge of mechanisms to prevent HIV and three misconceptions about HIV in this study. To assess the comprehensive HIV knowledge may need to use more items to improve its low effect on HIV testing.

Early sexual debut was the only significant predictor of the need-for-care factors (sexual risk behavior) using the Anderson model in this study. However, two other studies in Haiti and Ghana used symptoms such as genital discharge in the past 12 months for the need-for-care factors that positively predict HIV testing [32, 33]. Therefore, to increase the effect of need variables for positive uptake of HIV testing, more need-for-care variables such as symptoms may be explored in future studies.

Our study had some limitations. First, this study looked at adolescents and youth (15~24 years), limiting the generalizability to the broader population. Second, since we used secondary data, discrepancies from the selected samples may be a limitation. For instance, there was a wide variation in the selected samples, more so in females than males. Third, considering age and time, under-reporting past sexual history may have led to recall bias. Fourth, sexual behaviors and HIV testing are sensitive issues rarely discussed among adolescents, youth, and their parents. Thus, under-reporting of sensitive topics or over-reporting of insensitive topics may have incurred response bias. Fifth, regarding missing values, the efficiency of MIs could not be determined due to a lack of complete records in the data, and the ability to do so might have been reduced due to the high number of missing data [56].

## Conclusions

Our model testing results revealed a relatively low effect on uptake of HIV testing among youth (15~24 years) in The Gambia. Using Anderson’s Model of Health Service Utilization, the predisposing factors (socio-demographic and HIV knowledge) and the need-for-care factors (sexual risk behaviors) predict healthcare utilization services (HIV testing) in our study; however, only socio-demographic model explained most of the variance in HIV testing. The low effect of model testing could be related to the limited number of major variables selected for HIV knowledge and sexual risk behavior models. Thus, consideration for more variables is required for future studies.

## Supporting information

S1 Fig The behavioral model of HIV testing services among youth 15~24 years. (TIF)
