# Supplementary figures and images for: Predictors of HIV testing among youth aged 15–24 years in The Gambia
Source: PLoS One. 2022 Feb 18;17(2):e0263720. doi: 10.1371/journal.pone.0263720 (PMC8856544; doi:10.1371/journal.pone.0263720)

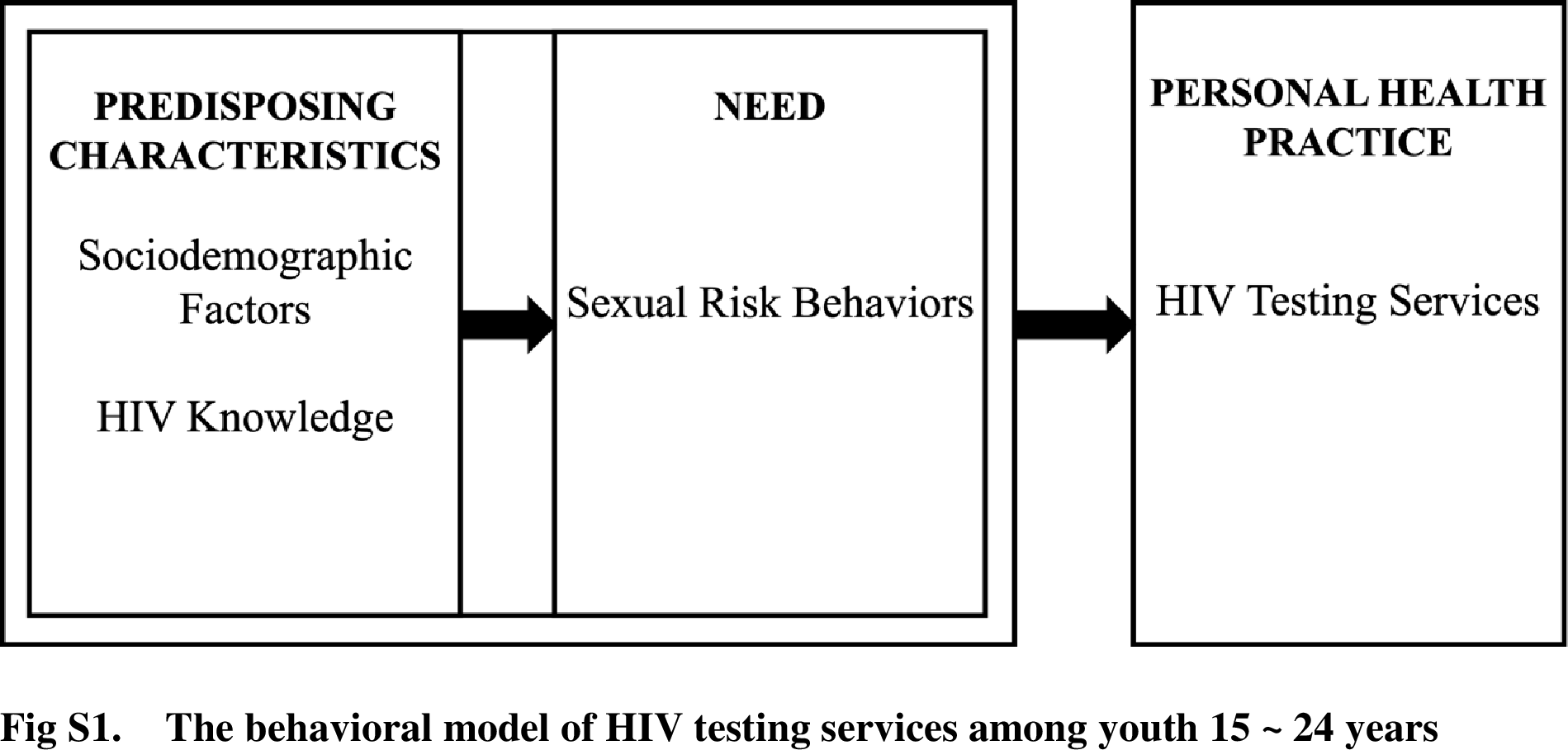

Supplement: S1 Fig — (TIF) [file pone.0263720.s001.tif]
